# Liver-Specific Knockdown of Class IIa HDACs Has Limited Efficacy on Glucose Metabolism but Entails Severe Organ Side Effects in Mice

**DOI:** 10.3389/fendo.2020.00598

**Published:** 2020-08-28

**Authors:** Nicole Ziegler, Suryaprakash Raichur, Bodo Brunner, Ulrike Hemmann, Manuela Stolte, Uwe Schwahn, Hans-Peter Prochnow, Christiane Metz-Weidmann, Norbert Tennagels, Daniel Margerie, Paulus Wohlfart, Maximilian Bielohuby

**Affiliations:** Sanofi-Aventis Deutschland GmbH, R&D, Frankfurt, Germany

**Keywords:** class IIa HDACs, type 2 diabetes, hepatocytes, gluconeogenesis, lipid metabolism, metabolic disease, hematopoiesis

## Abstract

Histone deacetylases (HDACs) are important regulators of epigenetic gene modification that are involved in the transcriptional control of metabolism. In particular class IIa HDACs have been shown to affect hepatic gluconeogenesis and previous approaches revealed that their inhibition reduces blood glucose in type 2 diabetic mice. In the present study, we aimed to evaluate the potential of class IIa HDAC inhibition as a therapeutic opportunity for the treatment +of metabolic diseases. For that, siRNAs selectively targeting HDAC4, 5 and 7 were selected and used to achieve a combinatorial knockdown of these three class IIa HDAC isoforms. Subsequently, the hepatocellular effects as well as the impact on glucose and lipid metabolism were analyzed *in vitro* and *in vivo*. The triple knockdown resulted in a statistically significant decrease of gluconeogenic gene expression in murine and human hepatocyte cell models. A similar HDAC-induced downregulation of hepatic gluconeogenesis genes could be achieved in mice using a liver-specific lipid nanoparticle siRNA formulation. However, the efficacy on whole body glucose metabolism assessed by pyruvate-tolerance tests were only limited and did not outweigh the safety findings observed by histopathological analysis in spleen and kidney. Mechanistically, Affymetrix gene expression studies provide evidence that class IIa HDACs directly target other key factors beyond the described forkhead box (FOXP) transcription regulators, such as hepatocyte nuclear factor 4 alpha (HNF4a). Downstream of these factors several additional pathways were regulated not merely including glucose and lipid metabolism and transport.

In conclusion, the liver-directed combinatorial knockdown of HDAC4, 5 and 7 by therapeutic siRNAs affected multiple pathways *in vitro*, leading *in vivo* to the downregulation of genes involved in gluconeogenesis. However, the effects on gene expression level were not paralleled by a significant reduction of gluconeogenesis in mice. Combined knockdown of HDAC isoforms was associated with severe adverse effects *in vivo*, challenging this approach as a treatment option for chronic metabolic disorders like type 2 diabetes.

## Introduction

Histone deacetylases (HDACs) are enzymes playing a major role in the epigenetic regulation of gene expression through the modification of histones. This highly conserved class of proteins thereby controls a wide range of different biological processes. HDACs and the counteracting histone acetyl transferases (HATs) acetylate and deacetylate lysine residues of histones, leading to an opening or condensation of chromatin that goes along with an increase or repression of transcription, respectively (1).

HDAC-mediated gene silencing is a multifactorial process that includes the recruitment of other epigenetic modifiers as for example histone- and DNA methyltransferases that further strengthen histone-DNA interactions and prohibit the transcription machinery from getting access to the DNA (2, 3). Furthermore, HDACs are also able to change the acetylation state of non-histone proteins including transcription factors, heat shock proteins and others (4, 5). By that, HDACs have a significant influence on the function and stability of their target proteins and regulate multiple cellular pathways and biological processes including proliferation, differentiation and survival (6, 7). As a consequence, HDACs are considered a promising therapeutic target for the treatment of a variety of diseases and several inhibitors are currently under investigation particularly in different types of cancer (8–10), but also cardiovascular and neurodegenerative disorders, inflammation and fibrosis (11–15). Vorinostat and romidepsin, for example, are pan-HDAC inhibitors being approved for the treatment of T-cell lymphoma. Due to their anti-inflammatory properties, vorinostat as well as trichostatin A (TSA) are additionally investigated in type I diabetes, multiple sclerosis and Alzheimer's disease (9, 16, 17). However, due to the broad biological function of HDACs in various tissues, pan-HDAC inhibitors have widely been associated with severe adverse effects including fatigue, diarrhea, hematological abnormalities and cardiotoxicity (18). Consequently, more recent approaches investigated compounds targeting only a subset of HDACs or even single isoforms such as nexturastat A, ricolinostat and PCI-34051, selectively inhibiting HDAC6 and HDAC8, respectively (19–21).

Based on their phylogenetic and functional properties, mammalian HDACs are categorized into four groups, whereby the second class is subdivided into class IIa (containing HDAC4, 5, 7, and 9) and class IIb (HDAC6 and HDAC10). Proteins of the different classes cannot only be distinguished by their catalytic activity, but also differ in their cellular localization. While class I HDACs (HDAC1, 2, 3 and 8) are ubiquitously expressed and show, except for HDAC3, mainly nuclear localization, class IIa proteins could be shown to shuttle between the nucleus and cytoplasm due to protein phosphorylation and have a tissue-specific expression pattern (22, 23). In recent years, increasing evidence demonstrates a substantial influence of HDACs on glucose and lipid metabolism, potentially qualifying pharmacological modulation of HDAC activity as an approach to counteract metabolic disorders including obesity and type 2 diabetes. Interestingly, several studies clearly connected the isoform-specific inactivation of class IIa HDACs with the reduction of hyperglycemia and insulin resistance as well as improved whole-body energy balance (24–27).

Treatment of metabolic dysfunction with HDAC inhibitors has failed in the past due to severe, and especially in case of chronic disease pathologies, intolerable side effects that are mainly due to the lack in tissue and isoform specificity. Therefore, the present study aimed to investigate if a tissue-selective inhibition of class IIa HDACs can exert beneficial effects on glucose metabolism paralleled by an acceptable safety profile. In order to achieve the highest possible degree of tissue- and isoform-specific inhibition, we investigated the potential of therapeutic siRNAs with a liver-specific nanoparticle formulation. We used these siRNAs to induce a single or combinatorial knockdown of HDAC4, 5, or/and 7 and analyzed the effects on hepatocellular glucose and lipid metabolism *in vitro* and *in vivo*. Furthermore, we conducted Affymetrix analyses to unravel novel, yet unknown interaction partners connected to these HDACs. Finally, we also investigated the safety of siRNA treatment by histopathological analysis of selected tissues.

## Materials and Methods

### siRNA Design, Synthesis and Formulation

For experiments with human hepatocytes, Silencer Select siRNA reagents were purchased from Thermo Fisher Scientific (HDAC4: s18839, HDAC5: s19464, HDAC7: s28335).

For mouse experiments publicly available siRNA design tools were applied from Invitrogen, Dharmacon and Whitehead to select 17 (Hdac4 and Hdac5) and 20 (Hdac7) different 19mer siRNA sequences, respectively (see Supplementary Figure 1). These sequences were synthesized either unmodified, with a light (UA, CA sequences) and with an extended (UA, CA, UG, CG sequences) 2′-O-methyl modification pattern of pyrimidine nucleotides. All siRNA sequences incorporated one 3′ terminal phosphorothioate linkage in the dT-dT overhangs. The following sequences were selected for *in vivo* studies: Hdac4: sense strand 5′-GGAAGAAAGuuuAAAcGAAdTsdT-3′, antisense strand 5′-UUCGUUuAAACUUUCUUCCdTsdT-3′; Hdac5: sense strand 5′-ccucAAGuGccGuGcGAAudTsdT-3′, antisense strand 5′-AUUcGcAcGGcACUuGAGGdTsdT-3′; Hdac7: sense strand 5′-GAAGAAAGcuGGAAAcAGAdTsdT-3′, antisense strand 5′- UCUGUUUCcAGCUUUCUUCdTsdT-3′ (capital letters = RNA, small letters = 2′-O-methyl RNA, s = phosphorothioate, dT = DNA-T).

For mouse *in vivo* experiments the liver-specific knockdown of target genes was achieved by intravenous injections of the siRNAs formulated in lipid nanoparticles (LNPs) based on Axolabs' proprietary cationic lipid XL-10 technology. Usage of this liposomal formulation has been demonstrated to result in a hepatocyte-specific knockdown of the siRNA target gene without significant effects in other tissues or cell types (28, 29).

### HDAC siRNA Screening in Mouse Hepa 1–6 Cell Line

Unmodified and modified HDAC4, 5 and 7 siRNA libraries were screened in murine Hepa 1–6 cells using 0.5 and 5 nM siRNA and Lipofectamine RNAiMAX transfection reagent (Supplementary Figure 1).

### HDAC Knockdown in Cultivated Human Hepatocytes

Frozen aliquots of upcyte® human hepatocytes (Upcyte Technologies) were used as described previously (30). Briefly, thawed cells were seeded in a density of 17,000 cells/cm^2^ in either precoated collagen type 1 96-well plates, for siRNA screening or 6-well plates for Affymetrix gene expression profiling. Transfection was performed using the Lipofectamine RNAiMAX Transfection Reagent (Invitrogen) following the manufacturers' protocol. In total, 1, 5 or 25 nM of non-silencing or HDAC 4, 5 or 7 siRNA either individually or in combination was delivered. After 48 h, cells were synchronized by replacing the standard medium by a serum- and BSA-free MEM (Thermo Fisher Scientific) for 4 h. Plates were then washed with pre-warmed PBS and freshly prepared gluconeogenesis induction medium (MEM containing 15 mM fructose, 10 mM lactate, 1 mM pyruvate, 5 mM L-alanine, 2 mM L-glutamine, 100 nM dexamethasone, 10 nM glucagon, 5 mM dibutyryl-cAMP, and 1 μM forskolin) was added to each well for additional 20 h. Transfected cells were harvested after 72 h and subsequently processed for RNA isolation.

### Gene Expression Analysis

Isolation of RNA from cell lysates was performed using the SV 96 RNA Isolation System (Promega) following the manufacturers' instructions. Isolation of RNA from mouse livers was done using Qiagen's RNeasy Mini Kit according to the supplier's protocol. Reverse transcription was performed using the high-capacity cDNA Reverse Transcription Kit (Applied Biosystems) and TaqMan® Assays (Life Technologies) were used for qRT-PCR in a LightCycler 480 instrument (Roche) or ABI Prism 7900 (Thermo Fisher Scientific). Raw values were normalized to GAPDH or RPL37A. Assay-IDs are listed in Supplementary Table 1.

### Affymetrix Gene Expression Profiling and Bioinformatics Analysis

Quantity and integrity of RNA isolated from siRNA-transfected upcyte cells was measured with an Agilent RNA 6000 Nano kit using an Agilent 2100 Bioanalyzer (Agilent Technologies Inc.). Amplification and labeling of probes and hybridization to Affymetrix chips was performed via a service provider (AtlasBiolabs) using a Human Genome U133 Plus 2.0 array. Bioinformatic analysis was performed using ArrayStudio (OmicSoft). Differentially expressed gene data were further interrogated on pathways and further causal relationship through the use of Ingenuity Pathway Analysis (Ingenuity, Qiagen) as described in Krämer et al. (31).

### Animal Studies

Adult, 8-week old female C57BL/6J mice were obtained from Charles River. Following a 2-week acclimatization period, mice were randomized into the respective treatment groups (*n* = 6–7 mice/treatment). During the experiment, mice had *ad libitum* access to filtered tap water and a standard rodent maintenance diet (Ssniff). The mice were group-housed at room temperature (20 ± 2.0°C) in an environmentally controlled SPF-animal facility on a 12 h light-dark cycle. All animal experimental procedures have undergone an ethical review and were approved by the internal animal welfare committee as well as by German government authorities.

Mice received five intravenous (i.v.) injections with either a control siRNA or one of the respective silencing siRNAs, i.e., Hdac4, 5 or 7 at a dose of 0.75 mg/kg, each. Another group received 5 injections with all 3 siRNAs simultaneously (again 0.75 mg/kg each with a combined siRNA dose of 2.25 mg/kg per injection). Mice received i.v. injections on experimental days 1, 4, 9, 15 and 22. All siRNAs were dissolved in PBS and the application volume was 5 mL/kg. On experimental day 11, 48 h after the 3rd i.v. injection, mice were fasted for 16 h to collect long-term fasting blood for analysis of blood glucose and plasma insulin. On experimental day 17, 48 h after the 4th i.v. injection, a pyruvate tolerance test was conducted. For this purpose, mice were fasted for 16 h and then received 2 g/kg sodium pyruvate (Sigma) by intraperitoneal (i.p.) injection. Multiple blood samples were collected from the tail tip for analysis of blood glucose for up to 120 min after the i.p. pyruvate bolus. On experimental day 24, 48 h after the 5th i.v. injection, mice were fasted for 6 h and sacrificed by cervical dislocation in deep isoflurane narcosis. Blood for EDTA-plasma was collected in chilled tubes pre-filled with EDTA and a protease inhibitor cocktail. All samples were collected, processed and stored as previously recommended to minimize pre-analytical variability of measurement parameters in metabolic rodent studies (32). For gene expression analyses, samples of liver tissue were immediately frozen in liquid nitrogen and stored at −80°C until further processing. For histopathological evaluation, organ weights were recorded with a precision scale and samples from liver, heart, kidney, spleen and pancreas were stored in 4% formaldehyde.

### Biochemical Analyses of Plasma Samples

Blood glucose was analyzed from hemolyzed samples (5 μl) of capillary blood added to 250 μl hemolysis reagent (Hengler Analytik) by the glucose-oxidase method using Roche Cobas 8000 systems and the GLUC2-reagent (Roche Diagnostics). Clinical chemistry parameters in terminal plasma samples were determined on a Cobas 6000 system equipped with a c501 clinical chemistry module. For ALT, AST, AP, GLDP, total cholesterol and triglycerides reagents ALTLP, ASTLP, ALP2, GLDH3, CHOL2, and TRIGL from Roche Diagnostics were used, respectively.

The following parameters were quantified using reagents from Fujifilm Wako Diagnostics: Free fatty acids were determined by HR Series NEFA-HR (2) reagents consisting of the following components: #999-34691 (Color Reagent A), #995-34791 (Solvent A), #991-34891 (Color Reagent B), and #993-35191 (Solvent B). Total ketone bodies were determined using an Autokit Total Ketone Bodies and the following reagents: #415-73301 (R1 set), #411-73401 (R2 Set). 3-hydroxybutyrate was specifically measured based on an Autokit 3-HB [Reagent components: #417-73501 (R1 Set), #413-73601(R2 Set)]. Additionally, acetoacetate concentration was calculated from the difference of total ketone bodies and 3-hydroxybutyrate concentrations. Plasma concentrations of total bile acids were assessed on a Beckman Coulter/Olympus AU680 autoanalyzer device using the TBA reagent from Randox Laboratories. Mouse insulin and glucagon were measured from plasma samples using chemiluminescent Mouse/Rat Insulin Kit (Meso Scale Discovery) and the Glucagon ELISA – 10 μl Kit (Mercodia), respectively.

### Histopathological Evaluation

Samples of liver, pancreas and heart were taken, fixed in 4% neutral buffered formaldehyde and embedded in paraffin. Sections were prepared following routine procedures, stained with Hematoxylin and Eosin (H.E.) and examined microscopically.

### Statistical Analyses

Control condition represents non-silencing siRNA that was performed equivalent to the targeted siRNA treatments in all experiments. PBS was used as a second control to exclude off-target effects but did not show differences relative to non-silencing siRNA and is for reasons of clarity not included in the figures. Data are shown as mean ± SEM. Statistical significance of *in vitro* experiments was analyzed by two-tailed Student's *t*-test. Statistical significance of data from *in vivo* studies was calculated using a nonparametric test for multiple comparisons (GraphPad Prism software version 8.0.2 for Windows). *P*-values < 0.05 were considered significant. A maximum of three significant digits are presented.

### Data Availability Statement

The Affymetrix data sets generated by siRNA mediated knockdown in human hepatocytes have been deposited in the Gene Expression Omnibus (GEO) public functional genomics data repository (https://www.ncbi.nlm.nih.gov/geo/) and are accessible through the GEO series number GSE154554 to help users query and download experiments and perform independently further analysis.

## Results

### Knockdown of HDAC4, 5 and 7 in Human Primary Hepatocytes Significantly Inhibits the Expression of Gluconeogenic Genes

**Figure 1:**
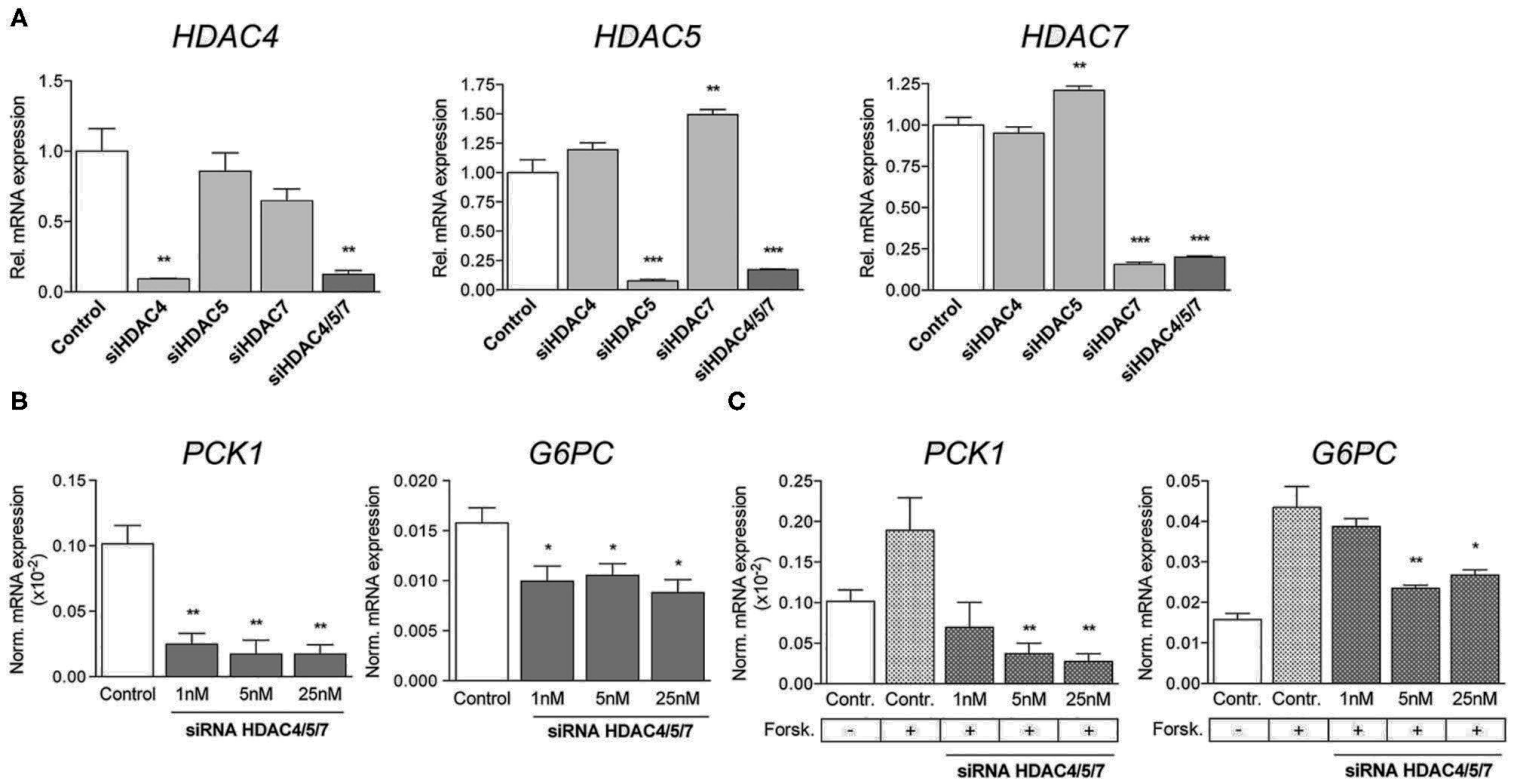
Simultaneous knockdown of HDAC4, 5 and 7 decreases gluconeogenic gene expression in human primary hepatocytes. **(A)** qRT-PCR for *HDAC4, HDAC5*, and *HDAC7* in primary human hepatocytes transfected with 5 nM non-silencing (control) or indicated siRNA pools. Expression levels are normalized and shown relative to control. **(B)** Normalized transcription of *PCK1* and *G6PC* in primary human hepatocytes transfected with 1nM, 5nM and 25nM HDAC4/5/7 siRNA **(C)** Normalized expression levels of *PCK1* and *G6PC* in primary human hepatocytes treated with 3 μM Forskolin and transfected with control siRNA or siRNA for HDAC4/5/7. Gene expression is shown relative to control. Columns represent mean ±SEM of four independent experiments. *p < 0.05, **p < 0.01, ***p < 0.001.

In order to evaluate the downstream effects of siRNA-mediated knockdown of HDAC4, 5 and 7, human hepatocytes were transfected with the indicated siRNAs either alone or in combination. Efficacy was confirmed by qRT-PCR and revealed an isoform-specific decrease in expression by ~90% with each individual siRNA and by ~80% with the combination of all three of them (Figure 1A).

The simultaneous knockdown of HDAC4, 5 and 7 was shown to abrogate the induction of gluconeogenic gene expression in mouse primary hepatocytes as well as in diabetic mice *in vivo* (24). Our present data replicate this finding in human hepatocytes, as transcription of phosphoenolpyruvate carboxykinase 1 (*PCK1*) and glucose-6-phosphate catalytic subunit (*G6PC*) was significantly decreased by about 80 and 40% relative to controls after transfection with different concentrations of siRNAs targeting HDAC4, 5 and 7 simultaneously (Figure 1B). Besides analyzing the potency of the siRNAs related to suppression of gluconeogenic genes under basal conditions, the effects were also investigated following the activation of HDACs by forskolin, an activator of adenylate cyclase stimulating nuclear translocation of HDACs. Forskolin increased the expression of *PCK1* and *G6PC* by 2- or 3-fold, respectively, and siRNAs at least partially abrogated this effect in a dose-dependent manner (Figure 1C). While *PCK1* expression was reduced below baseline levels, the effect on *G6PC* was less pronounced, indicating a higher sensitivity of *PCK1* promoter activation toward siRNA-mediated knockdown of HDAC4/5/7.

### Combined Knockdown of Class IIa HDACs in Human Hepatocytes Modulates Several Cellular Functions Beyond Gluconeogenesis

**Figure 2:**
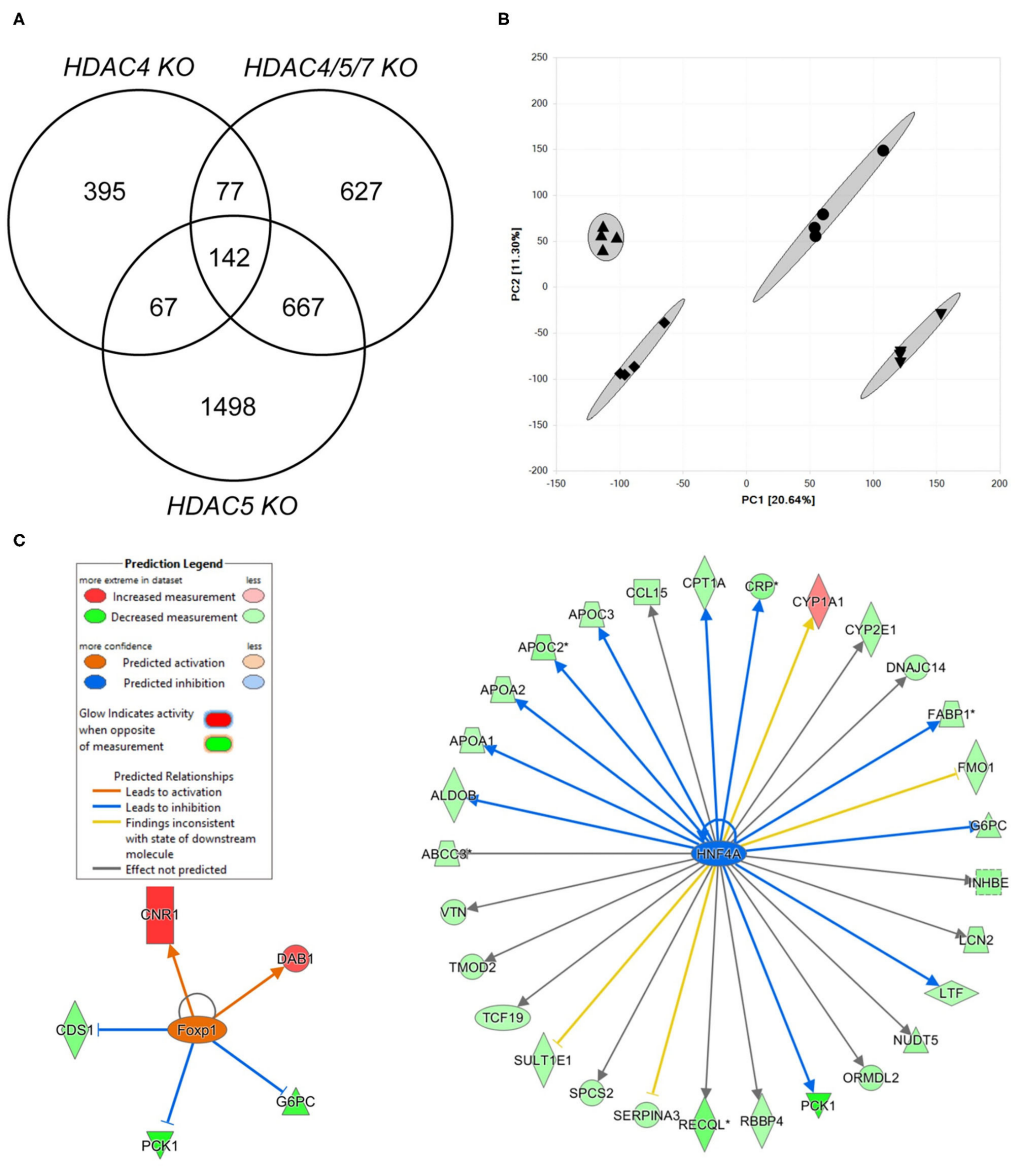
Transcriptomics analysis of human hepatocytes treated with HDAC4/5/7 siRNAs predict changes in lipid and carbohydrate metabolism that might be mediated by HNF4α. **(A)** Venn diagram showing the overlap in differentially regulated genes in human hepatocytes after combined knockdown of HDAC4/5/7 comparing to HDAC4 and HDAC5 knockdown. **(B)** Principal component analysis. **(C)** Visualization of analysis on upstream factors explaining the differentially gene expression. Shown are two identified upstream factors, FOXP1 and HNF4α, and how they modulate downstream genes differentially regulated by the combined knockdown.

In order to identify specificity of combined HDAC knockdown, we applied Affymetrix chip hybridization as more unbiased gene expression measurement. We transfected human hepatocytes with a combination of HDAC4, HDAC5 and HDAC7 siRNAs and compared these to either a scrambled siRNA or selected knockdowns of HDAC4 or HDAC5. More than 54,000 transcripts could be initially measured, 45,383 were mapped to known genes. GAPDH was equally expressed in all treatment groups, indicating that transcriptional activity in general is not affected by the siRNAs used in these experiments. Combined knockdown of HDAC4/5/7 resulted in significant expression change of 1,513 transcripts with an adjusted *p*-value < 0.05 and an absolute fold change >1.5 (Figure 2A). Single HDAC5 and HDAC4 knockdown as comparators resulted in 2,314 and 681 differentially regulated genes, respectively. The isoform-specific and the combined isoform knockdown groups all separated in a principal component analysis indicating a different gene expression pattern (Figure 2B). This coincides with the fact that only a few transcripts overlap in the Venn diagram. However, in all three knockdown groups, *G6PC* and *PCK1* were - among additional 140 transcripts - significantly downregulated. Next, we performed a more detailed analysis on pathways, upstream factors and networks making use of Ingenuity Pathway Analysis. Thereby, we focused first on which transcription factors and ligand-dependent nuclear receptors were predicted to be activated and inhibited in HDAC4/5/7 siRNA-treated primary hepatocytes relative to control (Table 1). Based on the regulation of five transcripts, *CDS1, CNR1, DAB1, G6PC*, and *PCK1*, this upstream analysis predicts forkhead box P1 (FOXP1) to be activated (Figure 2C). In addition, a couple of other transcription factors were identified to be inhibited, including ligand activated nuclear hormone receptors like RORα, cAMP responsive element binding protein like 3 (CREBL3), hepatocyte nuclear factor 4 alpha (HNF4α) and CCAAT enhancer binding proteins (CEBPA, CEBPE). A graphical representation of downstream target genes FOXP1 and HNF4α is provided in Figure 2C.

**Table 1:** Analysis of upstream factors predicted to be modulated by combined HDAC4/5/7 knockdown in human primary hepatocytes.

**Upstream regulator**	**Molecule type**	**Predicted activation state**	**Activation z-score**	***p*-value of overlap**	**Target molecules in dataset**
NRIP1	Transcription regulator	Activated	3.266	1.06E-05	AIFM2, APOA1, AREG, CDC6, CYP1A1, GYS2, HAS2, IDH3A, LCOR, PTGS2
MRTFB	Transcription regulator	Activated	2.227	1.87E-03	CXCR4, DDAH1, F2R, HPGD, LCN2, LTF, MPL, PCDH18, PDGFRA, PTGS2
FOXP1	Transcription regulator	Activated	2.180	7.62E-02	CDS1, CNR1, DAB1, G6PC, PCK1
CDKN2A	Transcription regulator	Activated	2.113	6.57E-02	ASF1B, BTG2, CASP3, CCNA1, CCR6, CITED2, CPA4, CRP, CXCL13, DHFR
NR0B2	Ligand-dependent nuclear receptor	Activated	2.039	1.19E-03	ABCB4, APOA1, APOM, CPT1A, CYP7B1, CYP8B1, G6PC, NR0B2, PCK1, PCK2
USF2	Transcription regulator	Inhibited	−2.042	1.23E-02	APOA2, APOA5, APOC3, CPT1A, PKLR, SERPINE1, THBS1, THRSP
TCF3	Transcription regulator	Inhibited	−2.043	9.64E-03	ASF1B, AZGP1, BHLHA15, BLNK, CASP3, CCNE2, CDCA3, CDK6, CIP2A, CTSV
CEBPA	Transcription regulator	Inhibited	−2.161	1.54E-08	ACSL1, ADH1B, AKR1B1, AKR1B10, AKR1C1/AKR1C2, APOC3, ASCL1, BTG2, CPT1A, CXCR4
RORC	Ligand-dependent nuclear receptor	Inhibited	−2.194	3.85E-06	APOA5, CCR6, CSF2, CYP2A6 (includes others), CYP2E1, CYP3A5, CYP7B1, CYP8B1, ELOVL6, ELOVL7
CREB3L3	Transcription regulator	Inhibited	−2.277	1.17E-06	APCS, APOA5, APOC2, CRP, CYP7B1, CYP8B1, G6PC, NFATC1, PCK1
RORA	Ligand-dependent nuclear receptor	Inhibited	−2.374	1.20E-05	APOA5, APOC3, APOE, CCR6, CYP2A6 (includes others), CYP2E1, CYP3A5, CYP7B1, CYP8B1, ELOVL6
CEBPE	Transcription regulator	Inhibited	−2.406	4.87E-03	CDK6, CTSV, IL1RN, LCN2, LTF, LYZ, PTGS2, SERPINB2
RUNX1	Transcription regulator	Inhibited	−2.464	3.82E-03	BAALC, BTG2, CSF2, CYB561, FAS, HBA1/HBA2, HMGA2, ID2, IL6R, IRF7
IRF2	Transcription regulator	Inhibited	−2.711	4.51E-03	ANG, C1QTNF1, CES1, CFB, CTSS, FGA, GBP1, IRF7, LCN2, MAPK6
TCF7L2	Transcription regulator	Inhibited	−2.914	2.14E-03	ACSL1, AKR1C4, APOD, ASPA, ATP8B1, CYP2E1, CYP3A7, ENPP4, EVI2A, EVI2B
EHMT1	Transcription regulator	Inhibited	−3.051	1.73E-03	ACSL1, BEST2, CP, EFNA1, HPD, LCN2, MMP28, PISD, PKP2, PPL
HNF1A	Transcription regulator	Inhibited	−3.403	3.77E-12	ABCC9, ADH1B, AFP, AHSG, AKR1C1/AKR1C2, AKR1C4, ALDOB, APCS, APOA2, APOC3
HNF4A	Transcription regulator	Inhibited	−4.152	1.99E-09	AASS, ABCC3, ABCC6, ACO1, ACSL1, ADH1B, AFP, AHSG, AKR1B1, AKR1C1/AKR1C2

Based on the array gene expression data, we performed an additional analysis on functions predicted to be activated or inhibited by combined HDAC4/5/7 knockdown (Table 2). A higher number of functions were predicted to be inhibited than activated, among them several glucose and lipid uptake and efflux functions.

**Table 2:** Analysis of functions predicted to be modulated by combined HDAC4/5/7 knockdown in human primary hepatocytes.

**Diseases or functions annotation**	***p*-value**	**Predicted activation state**	**Activation z-score**	**Underlying gene expression**
Aggregation of cells	9.96E-04	Increased	2.138	ADAMTS18, ADRA2A, CRP, DAB1, DMBT1, KIT, LTF, LYZ, THBS1, VTN
Accumulation of carbohydrate	1.42E-03	Increased	2.395	ABCB4, ABCC3, ADRA2A, APOA1, FABP1, SORD
Hepatic steatosis	2.94E-03	Increased	2.630	ABHD5, ADRB1, CNR1, CPT1A, CSPG4, CYP2E1, G6PC, LCN2, PCK1
Efflux of phospholipid	5.10E-08	Decreased	−2.131	ABCB4, APOA1, APOA2, APOC2, APOC3, FABP1
Flux of carbohydrate	5.63E-07	Decreased	−2.188	ABCB4, ABHD5, APOA1, CPT1A, FABP1
Efflux of lipid	1.08E-06	Decreased	−2.211	ABCB4, ABCC3, APOA1, APOA2, APOC2, APOC3, FABP1, PAPPA, VLDLR
Transport of lipid	1.19E-05	Decreased	−2.362	ABCB4, ABCC3, APOA1, APOA2, APOC2, APOC3, CPT1A, FABP1, PAPPA, SLC51B, VLDLR
Metabolism of carbohydrate	3.10E-05	Decreased	−2.424	ABHD5, ADRB1, AFF4, ALDOB, APOA1, APOA2, APOD, CPT1A, CREB3L3, CSPG4, CYP2E1, DCN, G6PC, GNG2, KIT, LTF, PCK1, PML, RGS4, SORD
Export of molecule	2.06E-04	Decreased	−2.474	ABCB4, ABCC3, ABHD5, APOA1, APOA2, APOC2, APOC3, FABP1, NUP107, PAPPA, VLDLR
Synthesis of carbohydrate	8.01E-04	Decreased	−2.035	ABHD5, AFF4, APOA1, APOA2, CREB3L3, CSPG4, DCN, G6PC, GNG2, KIT, LTF, PCK1, RGS4, SORD
Transport of molecule	1.24E-03	Decreased	−2.637	ABCB4, ABCC3, ABHD5, ADRA2A, APOA1, APOA2, APOC2, APOC3, ATP2B2, BHLHA15, CLCN5, CNR1, CPT1A, CYBRD1, CYP2E1, FABP1, G6PC, HBA1/HBA2, LCN2, LTF, NEDD9, NUP107, PAPPA, PML, SLC24A1, SLC51B, SULT1E1, THBS1, TNFSF15, VLDLR, XDH

### Targeting Hdac4/5/7 by siRNA Significantly Alters Hepatic Expression of Metabolic Genes *in vivo*

**Figure 3:**
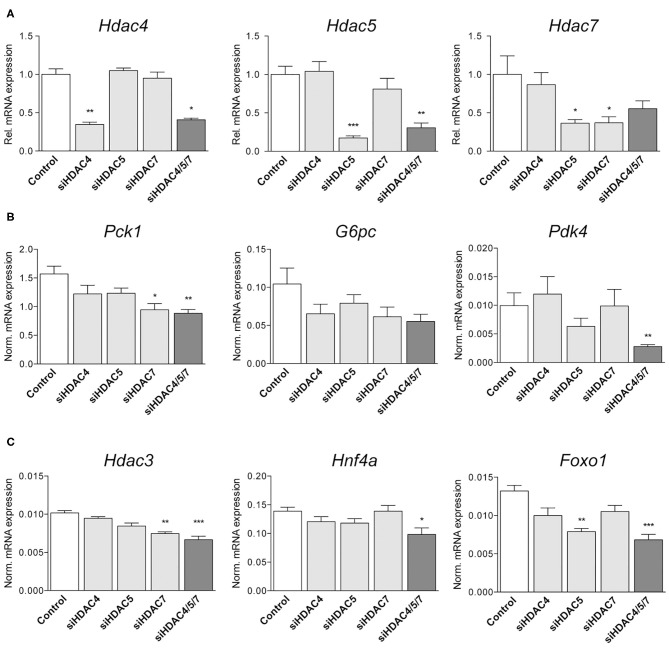
Knockdown of Hdac4, 5 and 7 significantly alters metabolic gene expression in mouse livers *in vivo*. **(A)** qRT-PCR for *Hdac4, Hdac5* and *Hdac7* in livers of C57BL/6 mice treated with 0.75 mg/kg non-silencing (control) or indicated siRNA pools. Expression is shown relative to control levels. **(B,C)** Normalized gene expression of *Pck1, G6pc*, and *Slc2a4 (Glut4)* as well as of *Hnf4a, Hdac3*, and *Foxo1* in livers of mice treated with control or indicated siRNAs. Gene expression is shown relative to control. Columns represent mean ± SEM of seven animals/group. Statistical comparisons were conducted using a nonparametric test for multiple comparisons; *p < 0.05, **p < 0.01, ***p < 0.001.

Following up on the *in vitro* findings, we analyzed whether similar correlations between knockdown of HDACs and the expression of selected genes could be found *in vivo*. For that, mouse siRNA libraries were first screened in murine hepatocytes (Supplementary Figure 1) and efficacies of selected siRNAs were confirmed. Subsequently, healthy mice were treated with either the most efficient single siRNAs (siHDAC4_8a, siHDAC5_12b, and siHDAC7_8a) or a combination of all three siRNAs. Knockdown efficiency was assessed by qRT-PCR in RNA isolated from liver. RNA concentrations as well as expression levels of the housekeeping genes were not altered due to the administration of any of our siRNAs, proving that gene transcription is not affected in general (data not shown). All single siRNA treatments resulted in isoform-specific knockdowns (Figure 3A). Although single isoform knockdown was slightly more effective, a knockdown of 60, 70 and 50% for Hdac4, 5 and 7 was obtained by the combined siRNAs inducing a triple knockdown, respectively (Figure 3A). Compensatory upregulation of one of the respective other HDAC isoforms could not be detected. However, we observed an additive effect in respect to the expression of downstream genes. Consistent with our *in vitro* data, expression of *Pck1* was significantly attenuated by the triple HDAC knockdown (Figure 3B) while the downregulation of *G6pc* did not reach statistical significance. Another enzyme being specifically correlated to metabolic disease and gluconeogenic gene expression, the mitochondrial protein pyruvate dehydrogenase kinase 4 (*Pdk4*) (33), was strongly inhibited in livers of mice treated with HDAC4/5/7 siRNAs. Furthermore, transcript levels of carnitine palmitoyltransferase 1A (*Cpt1a*), responsible for fatty acid transport, and the mitochondrially encoded cytochrome B (*mt-CytB*) were also significantly reduced with the triple knockdown (Supplementary Figure 2A). The same was true for *Hdac3*, whose recruitment is indispensable for the enzymatic activity of HDAC4, 5 and 7 (34–36), as well as for HNF4α and FOXO1 (Figure 3C), known inducers of gluconeogenic gene expression (37). Sirtuin 1 (SIRT1), a class III HDAC, is functionally dependent on NAD^+^ and acts in concert with PPAR Gamma Coactivator 1-Alpha (PGC-1α) to induce gluconeogenic genes (38). Consistent with our previous results, genes encoding for both proteins were strongest inhibited in livers of mice which received all three siRNAs, while the effects caused by single treatments were generally less pronounced (Supplementary Figure 2B).

### Combined HDAC Knockdown Does Not Significantly Affect Body or Liver Weights, Glucose Metabolism or Plasma Lipids in Healthy Mice

Body weights (all data in g; non-silencing control: 19.4 ± 0.2, siHDAC4: 19.3 ± 0.3, siHDAC5: 19.3 ± 0.3, siHDAC7: 18.9 ± 0.1, siHDAC4/5/7: 19.0 ± 0.7) and liver weights (all data in mg; non-silencing control: 899 ± 28, siHDAC4: 927 ± 30, siHDAC5: 1,002 ± 31, siHDAC7: 891 ± 18, siHDAC4/5/7: 855 ± 132) were not significantly different between the treatment groups. In addition, metabolic consequences going along with the siHDAC4/5/7-mediated transcriptional effects were assessed in parallel. Interestingly, the concentration of total ketone bodies was significantly reduced in mice treated with a combination of HDAC siRNA, likely due to the lowering of HO-butyrate (Table 3). Furthermore, a reduction in plasma total cholesterol was observed in mice with a triple HDAC4/5/7 knockdown as compared to isoform-specific knockdowns. Neither the concentrations of terminal plasma glucagon, nor plasma triglycerides, nor plasma free fatty acids, which may have an indirect influence on hepatic glucose production, differed between controls and single or combinatorial siRNA injection groups.

**Table 3:** Plasma parameters of mice treated with control, single HDAC4, 5 and 7 siRNAs and combined HDAC4/5/7 siRNAs.

	**Control**	**siHDAC4**	**siHDAC5**	**siHDAC7**	**siHDAC4/5/7**
ALT (U/l)	32.43 ± 1.7	37.29 ±3.06	29.14 ± 2.14	37.71 ± 2.25	30.71 ± 1.76
AST (U/l)	96.71 ± 5.94	115.43 ±17.39	74.0 ± 5.31	103.86 ± 12.18	104.57 ± 6.53
AP (U/l)	162.86 ± 5.78	182.86 ±6.27	165.14 ± 8.11	146.29 ± 6.84	142.0 ± 10.59^*^
GLDH (U/l)	9.8 ± 1.35	11.43 ±0.72	10.10 ± 1.08	7.64 ± 0.47	21.24 ± 2.87^***^
Acetoacetate (μM)	121.43 ± 17.05	107.0 ± 19.92	114.17 ± 15.39	80.0 ± 6.83	81.43 ± 13.4
HO-butyrate (μM)	765.29 ±73.32	631.43 ± 74.5	689.14 ± 102.8	486.86 ± 46.68	439.43 ± 59.94^*^
Ketone bodies (μM)	886.71 ± 86.96	738.43 ± 89.41	779.0 ± 104.4	566.86 ± 51.03	520.86 ± 72.18^*^
Cholesterol (μM)	1.83 ± 0.07	1.56 ± 0.04	1.96 ± 0.09	1.75 ± 0.08	1.46 ± 0.08^**^
Bile acids (μM)	6.53 ± 0.86	6.37 ± 0.85	5.04 ± 0.7	6.61 ± 0.86	5.78 ± 0.54
Glucagon (ng/l)	25.14 ± 5.62	18.81 ± 1.68	28.54 ± 3.88	27.58 ± 3.19	27.1 ± 5.0
FFA (mM)	1.01 ± 0.08	0.92 ± 0.04	1.04 ± 0.06	0.99 ± 0.06	1.09 ± 0.07
Triglycerides (mM)	0.83 ± 0.07	0.78 ± 0.04	0.9 ± 0.07	0.72 ± 0.06	0.82 ± 0.05

*Concentrations are shown as mean ± SEM of seven animals/group*.

* *p < 0.05*,

** *p < 0.01*,

*** *p < 0.001*.

*Bold lettering indicates significance*.

**Figure 4:**
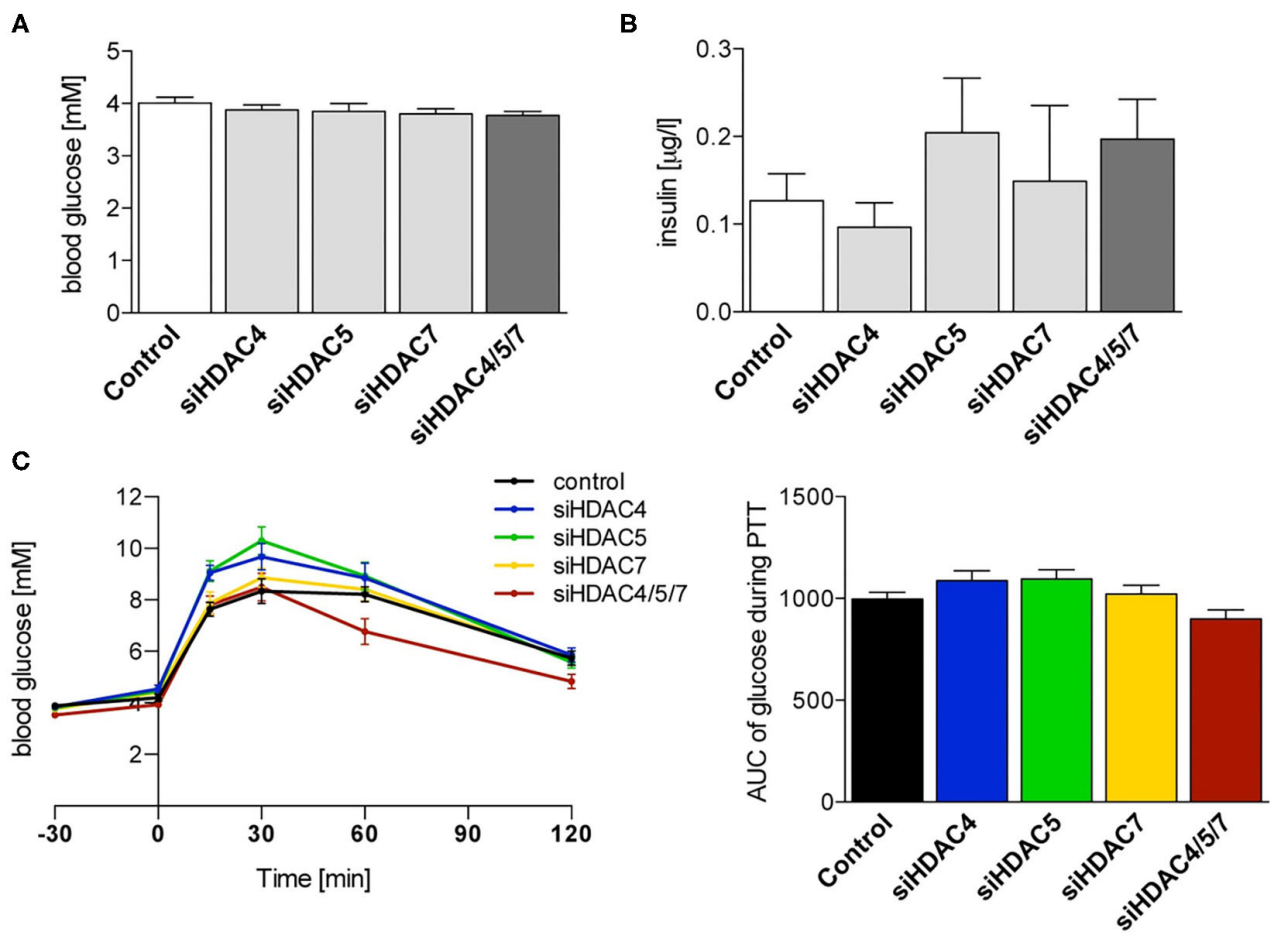
Suppression of Hdac4, 5 and 7 alone or in combination does not significantly affect glucose tolerance or fasting blood glucose and insulin levels in healthy mice. **(A)** Blood glucose and **(B)** plasma insulin concentrations in 16 h fasted C57BL/6J mice 48 h after the 3rd i.v. injection with control or indicated siRNA pools. **(C)** Blood glucose concentrations and calculation of the AUC during pyruvate tolerance test of the same mice as in **(A)**. Concentrations are shown as mean ± SEM of 5–7 mice/group. Statistical comparisons were conducted using a nonparametric test for multiple comparisons.

Despite the effective knockdown and significant changes in gene expression in the livers of siRNA-treated animals (Figure 3) and total ketone bodies, 16 h fasting blood glucose concentrations were not affected after three injections with any single or combinatorial HDAC siRNA treatment (Figure 4A). The same was true for insulin, which also did not significantly differ from control animals, neither with single siRNA treatment nor by the combination (Figure 4B). Of note, also the terminal, 6 h fasting blood glucose and plasma insulin concentrations in samples taken 48 h after the 5th injection did not change upon HDAC siRNA treatment. Blood glucose concentrations during a pyruvate tolerance test (PTT) appeared to be reduced in mice administered with all three siRNAs compared to all other groups. However, the calculation of the AUC did not mirror this deviation and the differences in AUC failed to reach statistical significance (Figure 4C). These data indicate that gluconeogenesis was overall affected by HDAC knockdown *in vivo*.

To focus on the investigation of potential side effects that might be accompanied by the inhibition of class IIa HDACs, additional blood parameters were analyzed (Table 3). The markers for hepatocellular injury, alanine aminotransferase (ALT) and aspartate aminotransferase (AST), were not significantly affected by the knockdown of HDAC4/5/7. However, concentrations of alkaline phosphatase (AP) were decreased while those of glutamate dehydrogenase (GLDH) were significantly elevated in animals treated with the combination of the three targeting siRNAs.

### Class IIa HDAC siRNA Knockdown in Mice Induced Morphological Changes in Spleen and Kidney

Due to the broad range of transcriptional pathways modulated by targeting of HDACs, the possibility of side effects beyond metabolic regulation may be a major issue.

**Figure 5:**
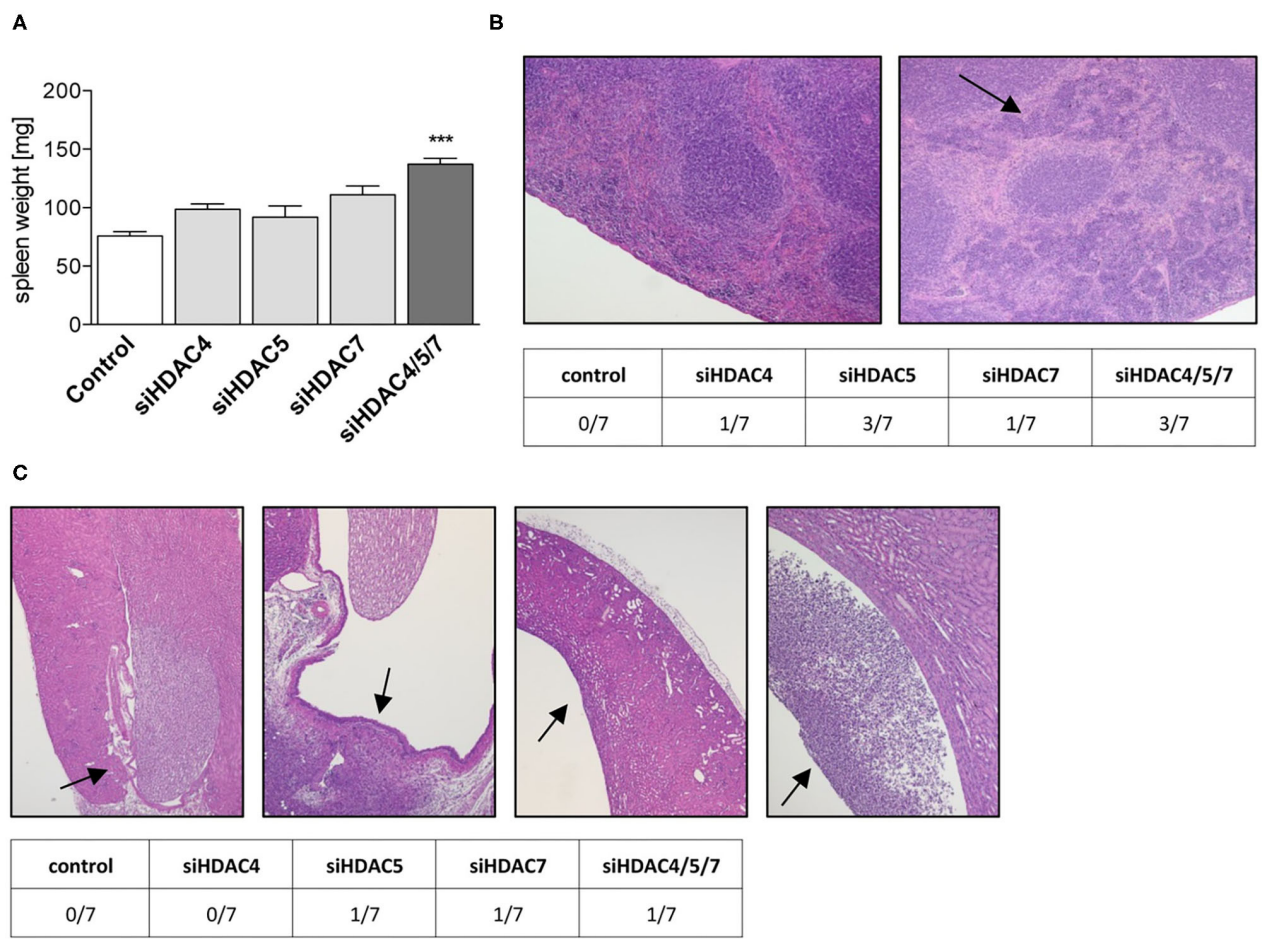
Liver-specific knockdown of Class IIa Hdacs in healthy mice lead to morphological changes in kidney and spleen. **(A)** Spleen weights of C57BL/6J mice after treatment with control or indicated siRNA pools. Bars represent mean ± SEM of 5–7 mice/group. Statistical comparisons were conducted using a nonparametric test for multiple comparisons; ***p < 0.001. **(B)** Representative images of H&E stainings of spleens showing normal (left) and increased grade 3 hematopoiesis (right, arrow) and distribution of both findings in the different treatment groups (table below). **(C)** Representative images of H&E staining of kidneys with normal pelvis and papilla (1st left, arrow) and with pelvic dilation and neutrophilic inflammation (2nd to 4th right, arrows). The table below the figure summarizes the appearance of the phenotype in different treatment groups.

In our model, microscopic examination of liver, pancreas and heart did not reveal any adverse findings in siRNA-treated animals compared to controls (data not shown). However, in all groups receiving a targeting siRNA, either single or in combination, an increase in spleen weight (significant for the triple knockdown) as well as in spleen hematopoiesis was observed (Figures 5A,B). In contrast, spleens of mice treated with the non-silencing control siRNA were without any adverse findings. Furthermore, potential target-mediated negative effects on kidney were detected, as signs of pyelonephritis were found in most of the HDAC treatment groups (Figure 5C).

## Discussion

In the present study, we demonstrate that the siRNA-mediated combined hepatic knockdown of class IIa HDACs 4, 5 and 7 has limited potential as a treatment option for metabolic diseases. The combined knockdown resulted in the already known liver transcriptional downregulation of key genes of gluconeogenesis like glucose-6-phosphatase and phosphoenolpyruvate carboxykinase. Several other transcriptional pathways, however, were also modulated, bearing the risk of additional unwanted effects. The minor, potentially beneficial contribution to glucose and lipid control observed in our study contrasted with severe side effects observed in spleen and kidney.

In general, impaired hepatic glucose production is a hallmark of type 2 diabetes (39). Consequently, inhibition of hepatic gluconeogenesis is regarded as one viable approach for treatment of type 2 diabetes as this mode of action is, for instance, described as part of the Metformin action (40) that is the most frequently used first line therapeutic for diabetes. With respect to hepatic class IIa HDAC inhibition, the observed clear reduction of gluconeogenic gene expression in cellular systems did not translate into sufficiently reduced gluconeogenesis in mice. Moreover, severe and likely target-mediated adverse effects were observed in spleen and kidney, being displayed as increased hematopoiesis and neutrophilic inflammation, respectively.

We could replicate in our studies data from other groups which have previously shown that the inhibition of class IIa HDACs reduces the expression of genes regulating gluconeogenesis (24, 26, 41). Initially, we confirmed these effects of our siRNAs *in vitro* in human primary hepatocytes. Besides a significant downregulation of *PCK1* and *G6PC* expression under steady-state conditions, the combinatorial knockdown of HDAC4, 5 and 7 was able to reduce gene expression following forskolin treatment, meaning when nuclear translocation and transcriptional function of HDACs is induced. Forskolin, however, is known to interfere with different signaling pathways by mediating the function of hormones and extracellular signals. For example, it is able to activate the nuclear receptors PXR and FXR that are involved in the modulation of hepatic energy metabolism via for example FOXO1 and FOXA2 (42, 43). Although we cannot exclude that those mechanisms interfere with our readout, the data clearly proves that our siRNAs are potent enough to repress gluconeogenic gene expression also under conditions in which transcription is increased. Based on these findings, the same siRNA sequences were used for further analyses.

Besides the now well documented effects on gluconeogenic genes, knowledge is still lacking about potential other genetic targets or downstream mediators especially under combined HDAC4/5/7 inhibition. We therefore analyzed the expression of more than 45,000 genes in human primary hepatocytes after combined knockdown of these HDACs by Affymetrix microarray technology followed by pathway analysis. We could show that the efficacy on gluconeogenesis genes may be mediated by upstream FOXP1 activation, but a plethora of transcription factors and modulators might additionally be involved in the mechanism.

Zou et al. showed that hepatic expression levels of FOXP1 are decreased in diabetic mice and, furthermore, modest hepatic overexpression of FOXP1 inhibited the expression of gluconeogenic genes (44). Mechanistically, they could demonstrate that FOXP1 physically interacts with FOXO1, thereby antagonizing its function as transcriptional activator of gluconeogenic gene expression. Consistent with these results, we demonstrated that *Foxo1* expression was significantly reduced by the combined knockdown of HDAC4, 5 and 7 in the liver.

However, FOXO1 function in hepatocytes is not only dependent on FOXP1, but also modulated by other factors such as PGC-1α that was shown to co-localize with HNF4α to induce the expression of genes including *Pck1* and *G6pc* while inhibition of PGC-1α reduces blood glucose concentrations and increases insulin sensitivity in diabetic mice (37, 45). Several additional candidate transcription factors and modulators were predicted in our analysis, consistent with additional literature findings that C/EBPβ and HNF4α and also CREB transcription factors induce the expression of gluconeogenic enzymes (46–49). One of these factors, HNF4α, plays a major role in coordinating hepatic gene expression and maintaining lipid homeostasis (50), explaining that its loss-of-function is associated with metabolic disturbances and liver disease (51, 52). The expression of HNF4α is controlled by epigenetic mechanisms and siRNA-mediated silencing of HDAC3 and 4 reduces its expression as well as target gene transcription in colon carcinoma cells (53). Our data suggest that a common mechanism regulating transcriptional activation also in the liver is likely. Significant downregulation of HNF4α was solely observed after the triple knockdown of HDAC4/5/7. Interestingly, this is the same treatment group in which also HDAC3 was decreased. A previous study demonstrated that both, HDAC3 and 4 isoforms interact with the transcription factor FOXO1 and that a simultaneous knockdown of HDAC3 and 4 attenuates its localization to gluconeogenic gene promoters (24, 26). Consistently, deficiency of FOXO1 resulted in a decrease in hepatic glucose production and reduced hyperglycemia in type 2 diabetic rats (54). Mechanistically, combined HDACs Class IIa knockdown seems to go clearly beyond target-specific inactivation of dedicated genes but rather intervenes in regulatory co-expression networks. In line with that conclusion, the combined knockdown of HDAC4/5/7 may not only restore epigenetic patterns, but could also intervene with the activity of non-histone proteins and thereby alter fundamental signaling cascades in line with previous suggestions (5).

Hepatic expression of *Hnf4*α, *Foxo1* as well as *Pgc-1*α was significantly lower in HDAC4/5/7-treated mice compared to controls, however the impact on gluconeogenic gene expression *in vivo* was limited. Results from pyruvate tolerance tests in overnight fasted mice, being used as a dynamic surrogate test to assess the gluconeogenic capacity *in vivo* (55), clearly showed that knockdown of individual HDAC isoforms 4, 5 or 7 does not affect gluconeogenesis to a significant extent in healthy mice. If, at all, only the triple knockdown of all three HDAC isoforms showed a minor effect that however, did not reach statistical significance compared to controls. Thus, it seems that the selective hepatic knockdown of HDACs in healthy mice can significantly reduce the hepatic expression of selected genes controlling gluconeogenesis and decrease moderately ketone body production. However, overall, these two effects do not sum up sufficiently to affect whole-body gluconeogenesis control to a measurable extent. In line with our data from the pyruvate tolerance tests, also fasting blood glucose and plasma insulin concentrations were not significantly affected by siRNA treatment.

With respect to lipid metabolism, the Affymetrix data from human primary hepatocytes suggest defects in the transport and especially the efflux of phospholipids upon simultaneous knockdown of HDAC4/5/7, which, moreover, was predicted to be caused by the decrease in *Hnf4*α expression. Consistently, it was shown by Hayhurst et al. that mice lacking hepatic expression of HNF4α exhibit an accumulation of lipids in the liver while the concentration of serum triglycerides and cholesterol is decreased and bile acids are elevated (50). We did neither observe changes in plasma free fatty acids and triglycerides, nor differences in liver weight, which is usually elevated in mice with altered lipid homeostasis, e.g., in hepato-steatosis. As, moreover, the detailed histopathological evaluation did not reveal any signs of lipid accumulation, we assume that liver lipids are not affected by the knockdown of class IIa HDACs. However, the concentrations of plasma cholesterol were significantly lower in the triple knockdown group so that a secondary effect of HDAC knockdown appears to be plausible. Although we cannot exclude the possibility that an extended treatment duration would have led to increased liver lipids, overall, the plasma lipid data confirm that the metabolic effects *in vivo* were rather weak.

Irrespective of the limited potency of siRNA-mediated silencing of HDAC4/5/7 on glucose and lipid metabolism in healthy mice, the histopathological analysis of key organs revealed detrimental side effects in mice with a single or combinatorial knockdown of HDACs. Interestingly, the adverse findings were detected in spleen (increased hematopoiesis) and kidney (chronic purulent pyelonephritis), but not in the target organ for the knockdown, i.e., the liver.

Previous studies demonstrated that the specific siRNA formulation technology used in our studies delivers specifically siRNA to the liver (29, 56). Consequently, the serious side effects in other organs may be a consequence of changes originating in the liver. A direct immune response to siRNAs nano particles is very unlikely as our formulated siRNAs harbor a 2′-O-methylation, which could be shown to minimize immune stimulation compared to unmodified siRNA (28). Moreover, the side effects did not occur in a control group with unscrambled siRNA nanoparticles. Thus, we assume a disturbed immune response that is induced by the hepatic knockdown of this class of HDACs and then spills over in a paracrine manner to other more immune sensitive organs. Several groups investigated the effects of HDACs as well as small molecule inhibitors in respect to immune reaction and could demonstrate that class IIa HDAC isoforms are involved in different immunological processes including macrophage activation as well as B- and T-cell development (57–59). Such effects could also occur locally in the liver on resident immune cells. Overall, the pathological mechanism for these alterations in spleen and liver remains unclear and subject of further studies.

One may argue that the lack of efficacy on glucose metabolism in siRNA-treated mice is masked by the animal model used in this study, i.e., healthy mice without any impairment in glucose metabolism and without elevated hepatic glucose production *per se*. While we certainly cannot exclude that the efficacy of a liver-selective class IIa HDAC knockdown on improving glucose metabolism and reducing gluconeogenesis might have been more pronounced in mouse models of type 2 diabetes, we decided not to replicate our siRNA approach in such a disease model. This decision was mainly driven by the unexpected and undesirable histopathological findings with siRNAs targeting the HDACs, which likely would have also occurred in mouse models of type 2 diabetes. Importantly, the observed serious safety concerns *per se* excluded a further development of this approach for metabolic diseases and thus, for ethical reasons, we did not conduct any further metabolic *in vivo* studies with these siRNAs. Another potential reason for the limited *in vivo* effects on glucose metabolism could have been an insufficient knockdown efficacy with a too high residual expression masking effects on metabolic downstream parameters. Clearly, a higher dosage of siRNAs could have led to a more pronounced knockdown and, consequently, to more pronounced metabolic effects. However, again and in light of the histopathological findings, we decided to not follow this approach further as the detected severe side effects on an organ level would not be acceptable in any treatment regimen for type 2 diabetes and thus the therapeutic approach *per se* has to be considered as inappropriate.

In conclusion, our data provide evidence that class IIa HDACs directly target and thus regulate the expression of *Hnf4*α, *Foxo1*, and *Hdac3* in the liver, thereby modifying gene regulatory mechanisms mediating glucose and lipid metabolism and transport. Simultaneous knockdown of HDAC4/5/7 significantly decreased gluconeogenic gene expression *in vitro*. Nevertheless, although achieving valuable efficacy of the siRNAs, metabolic effects of liver-selective delivery were limited and did not affect whole-body gluconeogenesis in healthy mice. Instead, adverse effects in spleen and kidney were observed. Thus, potential beneficial effects of the liver-specific inhibition of selective HDAC isoforms via therapeutic siRNAs did not outweigh potential safety concerns, illustrating that the development of class IIa HDAC inhibitory siRNA molecules for chronic treatment of metabolic disorders is likely not a viable approach. With respect to treatment of other life-debilitating conditions, such as cancer, potential benefits must be carefully balanced against potentially harmful off-target effects.

## Data Availability Statement

The Affymetrix data sets generated by siRNA mediated knockdown in human hepatocytes have been deposited in the Gene Expression Omnibus (GEO) public functional genomics data repository (https://www.ncbi.nlm.nih.gov/geo/) and are accessible through the GEO series number GSE154554 to help users query and download experiments and perform independently further analysis.

## Ethics Statement

The animal study was reviewed and approved by Internal animal welfare committee as well as by German government authorities.

## Author Contributions

NZ wrote the manuscript, performed *in vitro* and *ex vivo* experiments and analyzed data. SR performed *in vitro* experiments and analyzed data while still being an employee of Sanofi-Aventis. BB designed and selected the siRNAs, performed *in vitro* experiments, analyzed the data, and wrote the manuscript. UH supported the toxicological assessment of the concept. MS did the histopathological analyses of tissues. US and H-PP analyzed plasma samples. CM-W isolated RNA and performed gene expression analyses. NT wrote the manuscript. DM analyzed Affymetrix data. PW performed *in vitro* and *ex vivo* experiments, analyzed data, performed pathway analyses, and wrote the manuscript. MB conducted *in vivo* studies, analyzed data, and wrote the manuscript. SR, BB, NT, PW, and MB oversaw the project. All authors contributed to the final editing of the manuscript.

## Conflict of Interest

NZ, SR, BB, UH, MS, US, H-PP, CM-W, NT, DM, PW, and MB are (or were during the time of project completion) employees and shareholders of Sanofi. SR is presently an employee of Evotec International GmbH, Germany. Employment at Sanofi-Aventis or Evotec did not affect interpretation of data or experiments.
