# Supplementary material for: Liver-Specific Knockdown of Class IIa HDACs Has Limited Efficacy on Glucose Metabolism but Entails Severe Organ Side Effects in Mice
Source: Front Endocrinol (Lausanne). 2020 Aug 28;11:598. doi: 10.3389/fendo.2020.00598 (PMC7485437; doi:10.3389/fendo.2020.00598)
Supplement: Supplementary file 1 [file Table_1.docx]

| **Gene name** | **Assay ID** |
| --- | --- |
| Human |  |
| G6PC | Hs02560787_s1 |
| HDAC4 | Hs01041638_m1 |
| HDAC5 | Hs00608366_m1 |
| HDAC7 | Hs00248789_m1 |
| PCK1 | Hs01572978_g1 |
| RPL37A | N/A (self designed) |
| Mouse |  |
| Cpt1a | Mm01231183_m1 |
| Foxo1 | Mm00490671_m1 |
| G6pc | Mm00839363_m1  Mm04207417_m1 |
| Hdac3 | Mm00515916_m1 |
| Hdac4 | Mm01299557_m1 |
| Hdac5 | Mm01246076_m1  Mm00515929_m1 |
| Hdac7 | Mm00469527_m1 |
| Hnf4a | Mm01247712_m1 |
| mt-Cytb | Mm04225271_g1 |
| Pck1 | Mm01247058_m1 |
| Pdk4 | Mm01166879_m1 |
| Ppargc1a | Mm01208835_m1 |
| Rpl37a | N/A (self designed) |
| Sirt1 | Mm01168521_m1 |
| Gapdh | Mm99999915_g1 |

Supplementary table 1
