# Supplementary figures and images for: Liver-Specific Knockdown of Class IIa HDACs Has Limited Efficacy on Glucose Metabolism but Entails Severe Organ Side Effects in Mice
Source: Front Endocrinol (Lausanne). 2020 Aug 28;11:598. doi: 10.3389/fendo.2020.00598 (PMC7485437; doi:10.3389/fendo.2020.00598)

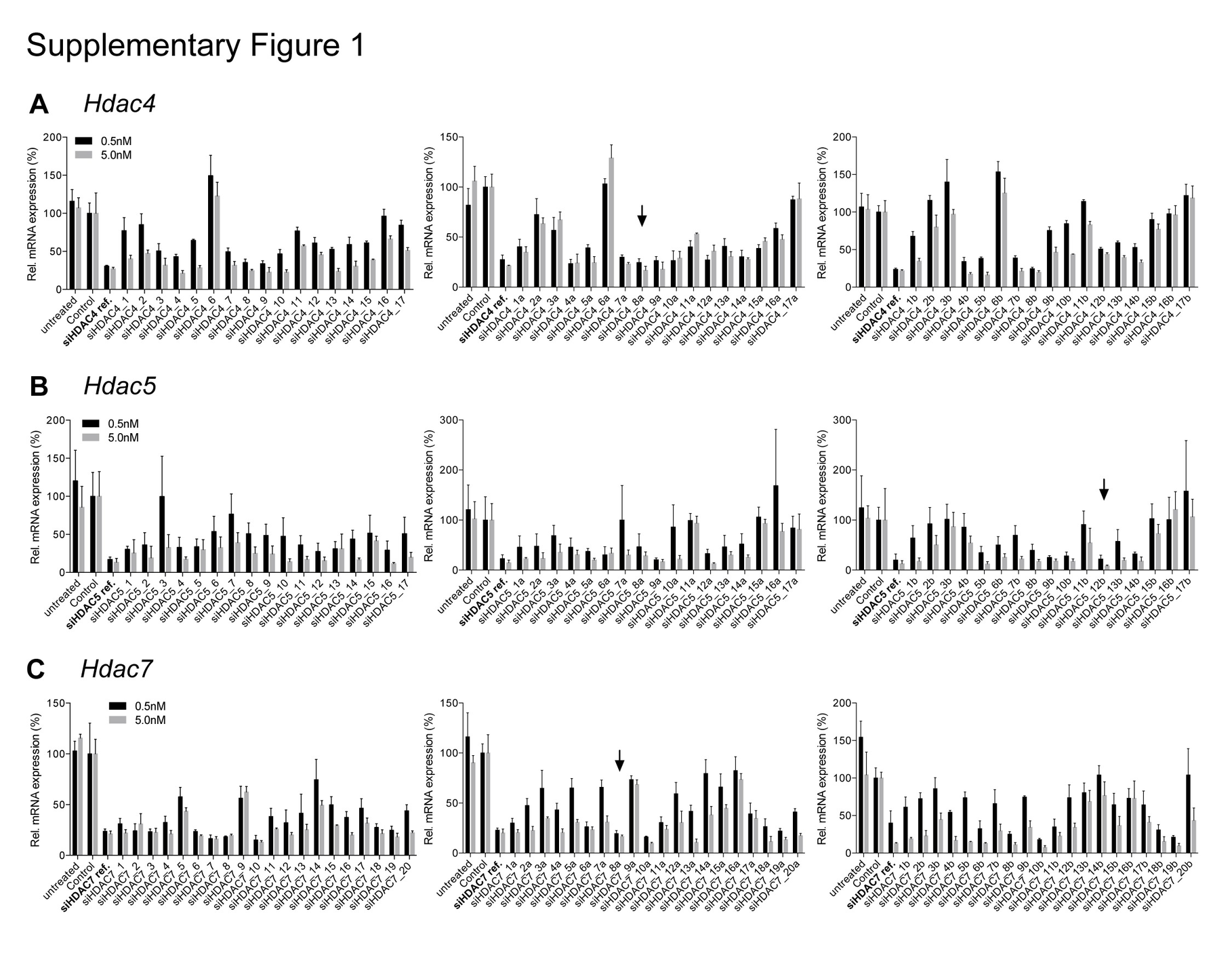

Supplement: Supplementary file 2 [file Image_1.TIF]

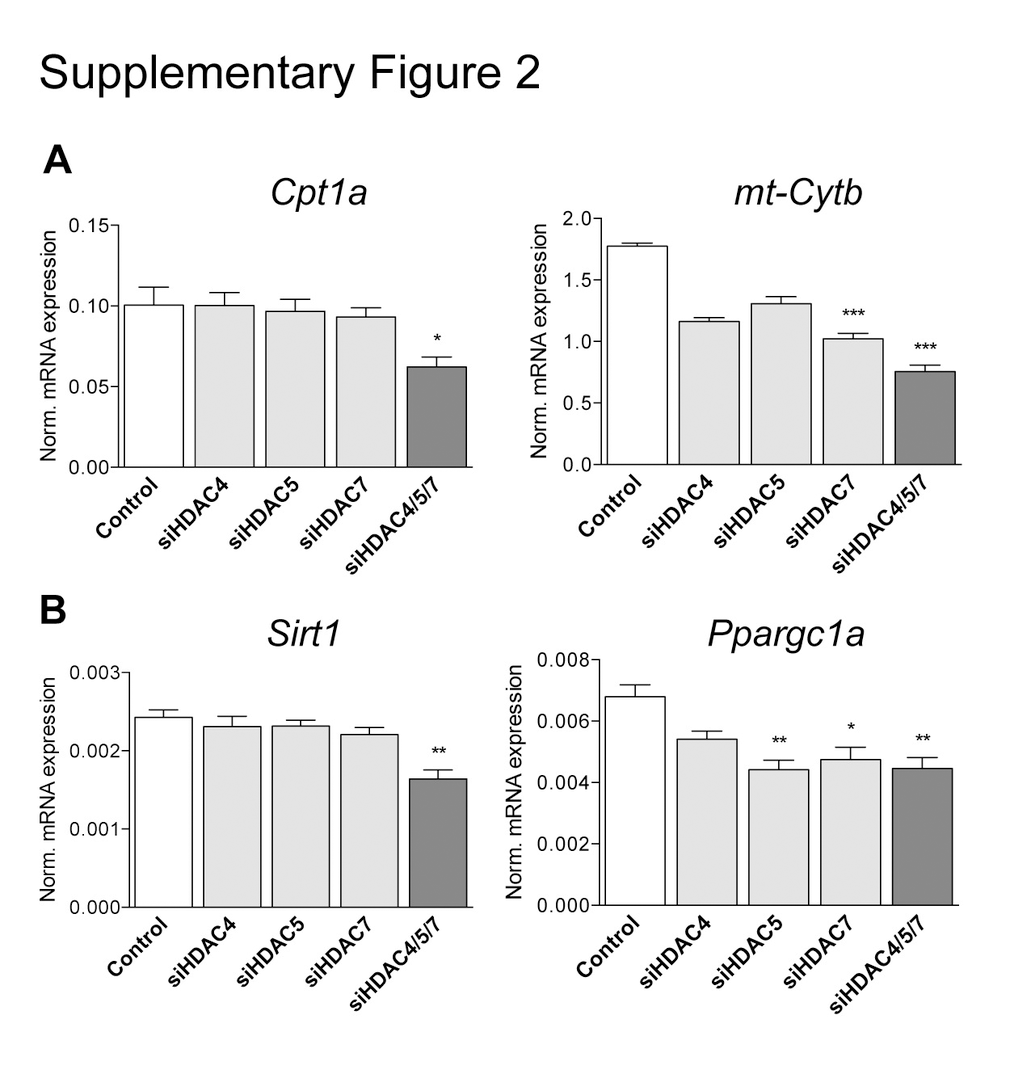

Supplement: Supplementary file 3 [file Image_2.TIF]
